# The Last Sentiments of Suicides

**Published:** 1851-10-01

**Authors:** A. Brierre de Boismont


### THE LAST SENTIMENTS OF SUICIDES.

BY DR. A. BRIERRE DE BOISMONT.

*(Translated from the Author's MSS.)*

### III. Mixed Sentiments.

This last class contains the analysis of twenty-one varieties of sentimental expressions, comprising 316 cases (256 men, 60 women). At the first glance, the sentiments expressed in these writings appear neither good nor evil; they refer to the person's position, or contain certain special directions; yet, on closer examination, some of the sentiments expressed will be found to appertain to the first class, and others to the second; we have, therefore, thought it advisable to give them the designation, mixed. It will be easily understood that such a classification cannot be very rigorous, nor, indeed, does the nature of the subject admit of it; for the various alliances of the moral sentiments cannot be clearly separated or defined. Nevertheless, we have adopted this kind of division for convenience of reference.

This third class is divided into eight sub-sections, based on the peculiar character of the sentiments expressed.

1st Sub-section. *State of the mind in relation to the act; assert that they are in full possession of their reason; that they are the authors of their own death; that no other is to blame; testamentary bequests; sang-froid.*—The imputation of insanity, indiscriminately applied to all suicides, is abundantly contradicted by history and observation. The following extracts from our store of letters, show that persons may destroy themselves without manifesting any signs of aberration of intellect; that they accomplish the act coolly, calmly, deliberately, and in a rational manner. We find this condition clearly marked in 57 cases (48 men, 9 women). We will quote from some of their letters:—

"After working hard for fifty years, and having saved nothing,—being old, decrepit, and destitute, yet unwilling to enter those boxes which they call 'asylums for the aged,'—we prefer to die. It is the advice which Montaigne gives on such an occasion, and it is the practice followed by Atticus—rich, but old and suffering. There is not a shadow of folly in our resolve."

"They say it is no proof of courage to kill oneself—that it is madness; very well! I, being within a hand's-breadth of death, assert the contrary; of sound mind and body, seeing that the carbonic acid gas does not produce its effect with sufficient rapidity, I have twice got up to fan the charcoal, and give it greater force. I have my full reason; an old soldier like me does not fear death, but I ought to have died on the field of battle; what a pity that I did not find a tomb at Essling, where my regiment covered itself with glory."—"My dear son, when you get these lines I shall have ceased to live; God only knows what a struggle I have had to endure life up to this day, so as to be of service to you and your sister. Be her protector, for she will have great need of you. The thought of her torments me in my last hour."—"Monsieur M— is the author of my disaster; if he had consented to take back the farm in Berry, the management of which was ruining me, I could have got my head above water again. After passing several days without any means of subsistence, almost without bread, I accomplish my design in a lodging-house frequented by workmen, who are now gone to their labour."—"To M. —, the Commissary of Police, who will have me taken somewhere. The loss of my situation, the death of my eldest daughter, my debts and poverty, are the chief causes of my fatal determination. I could not endure the sight of my wife and seven children exposed to want, and sometimes without food, without experiencing a thousand tortures. Moreover, my unfortunate position had made all my clients leave me. Infirm, invalid, without means, I could not hope to succeed in so unequal a struggle, and had nothing left me but death. I may, perhaps, be accused of want of courage; but where, I ask, is the folly, when one is reduced to such an extremity? My parents did nothing for me; and if I had followed the advice of my uncle who brought me up, I should not be in this position."—"I earnestly warn you, my dear son, not to follow my example. Burn all my books without opening them; this is my last request."—"I have a disease which requires a painful and expensive surgical operation. I do not believe myself capable of supporting the pain, and I cannot afford the outlay. My position in life does not admit of my going into the hospital, so I adopt a certain cure, which is to asphyxiate myself. I have chosen this locality in preference to my own room, as being more convenient, and giving me greater freedom of action. I desire that my body may be taken to the medical school." This is succeeded by testamentary directions, which evince perfect presence of mind. "At least," writes a young woman, "do not mourn for me; I am happier dead than living; I have rid myself of a painful existence, whilst you, who are wretched, condemn yourself to further suffering. I freely pardon the injuries inflicted on me, but I could no longer live like that. I request that my body may not be opened. Mine is not an insane deed—not the impulse of despair—but a rational act. Spend only the sum necessary for burying me in the simplest manner."

The foregoing examples suffice to explain the disposition of mind in the writers; the others are merely a repetition of the same sentiments, the motives being those already indicated. It should be noticed that the majority of these letters are in a fine steady handwriting; twenty-six are very well written; many present no erasures; some are very lengthy. There are some in English, German, Italian, and Portuguese. Six are headed—"*One hour before my death.*" Some have been cut short by the fall of the writer, chiefly from asphyxia. The most curious case of this kind is that already quoted, of the man who registered his suffering from minute to minute.

The examination of these manuscript notes is the best refutation of the idea that a certain amount of delirium, and an appreciable disorder of the mind, always attend the supreme hour of suicides. But it is not merely the handwriting of these letters which shows what perfect command many suicides have over themselves at their last hour, but also the sentiments which they express. A student writes to a physician, related to him,—"I afford you an admirable opportunity to pursue your phrenological studies; I am about to suffocate myself. You will even render me a great service; for, in case of resurrection, I shall be curious to learn whether I had the bump of suicide. I wish you to understand that I have nothing the matter with me, and that I destroy myself with the most perfect *sang froid.* There can be no balancing between death and disgrace."

Among 4595 individuals who destroyed themselves, 85 (63 men, 22 women) made testamentary arrangements. The majority of these documents evince great *sang froid,* a strong will, and perfect lucidity of intellect.

These wills are dictated under the influence of ideas common to all men at such a crisis. Some leave their fortunes and possessions to their relatives, to the persons they love, to those who have been kind to them, or taken care of them during an illness; others disinherit those who have offended them. Many designate certain objects which do not belong to them, with the request that they may be restored to the owners. One of the most striking of these histories is that of a man who declares that he has burnt every legal title or security of a considerable property, with the view of depriving his wife of the enjoyment of it, she having been the torment of his life.

Among the most singular wills is that of a man who destroyed himself in 1832, in which he asserts that he has left a fortune of 12,000*l.* in trust to three faithful friends, who will render it to his only son in 1848, should the said son be living at that date. He says, that the precautions he has taken leave him no inquietude about it. The most careful inquiry failed to elucidate this affair. A young lady bequeaths all she died possessed of to her brother, in order that he may not follow her example, but be able to marry the person he loves. "I give everything upon this bed to the person who will inter me; I am sorry it is not more, but it is all I have."

It has been stated that those who have failed in one attempt to commit suicide, rarely make a second, unless they are decidedly insane. In fourteen instances this was not the case, and some of these individuals persevered in their project even when mortally wounded. A man discharged a loaded pistol into his mouth, and, although horribly mutilated, as death arrived too tardily, and he had not strength to load the pistol again, he managed to drag himself to the window, and drop into the street. A workman threw himself under the wheels of a cart filled with paving-stones, and when it had passed over him, cried out, "I wished to be killed, and I am merely wounded; if I could get up I would go and drown myself;" a few minutes after he expired. A soldier withdrew into a wood, and discharged a pistol point blank into his chest; after that he walked some distance to an inn, and took a room. He shut the door and windows, and was preparing to suffocate himself, when weakness obliged him to throw himself on the bed. In a short time he was dead. Those who have failed in accomplishing their purpose, commonly entreat the persons who are taking care of them to leave them alone. "Let us die in peace! We wish to die! Our only regret is that we have failed."

Among many facts of this kind, one which we owe to the kindness of the late Dr. Sarlandière, leaves no doubt as to the perfect composure and tranquillity of mind sometimes shown by suicides, even after the perpetration of the act. In 57 cases which we have examined specially, in relation to the integrity of the reason, 20 (16 men, 4 women,) declared that they were alone the authors of their death, and, therefore, no other person should be accused. Many even exculpate certain persons who, by reason of evil relations with them, might possibly be suspected of having a concern in their death.

*Confusion of mind—their ideas are troubled.*—Opposed to the foregoing documents, which testify to the *sang froid* and presence of mind of the writers, are others which evince trouble and perturbation of mind.

This series, composed of 55 pieces, (43 men, 12 women,) presents three different shades or degrees, dependent on the nature of the mental disturbance, whether due to old standing alienation, to temporary delirium, or simple exaltation at so dreadful a moment.

Although insane persons who commit suicide in lunatic asylums rarely leave any writing behind them, those, on the contrary, who have been permitted to live at large and mix in society sometimes make known the motives which animate them. The letters, which present unmistakable evidences of insanity, are thirty-four in number. And here we have a confirmation of the opinion, often set forth by us, that the official returns of the number of insane persons being deduced from the numbers actually treated in public and private establishments, are of necessity imperfect and incorrect, and afford merely approximate information as to the real ratio of insanity to the population at large. The proportion of suicides in whom previous aberration of mind could be traced being 1013 cases, or about 25 per cent. of the total number, nearly the whole of whom were at liberty at the time the fatal deed was committed, is a proof that there exists a large amount of insanity which is not officially recognised.*

The motives assigned by the persons classified in the first series clearly indicate the condition of their minds. Here, for instance, are their expressions—"My disappearance from my place must be attributed to a sudden attack of insanity, with which malady my father was afflicted, and of which he died. I was about to get married, and live happily. This attack of madness, which they tell me lasted ten days, but which I know nothing about, is my death-warrant."—"The insulting observations continually made around me, and the calumnies of my neighbours, are the cause of my death."—"For some time past I have been sensible that I no longer possess sufficient capacity for my employment: they have an eye on me: I shall some day make a terrible blunder, so it is better to escape from this overwhelming perplexity at once."—"I have committed an odious crime, and I am incessantly pursued by a voice reproaching me, and never permitting me an instant of repose."—"Go thou and do likewise! pitch thyself over; we have offended God, and we shall be eternally damned."

A considerable number of these individuals say that they are fearful of going mad—that they have lost their reason—that their ideas are troubled —that they feel their mind failing.

We have already signalized the fact of the moral contagiousness of insanity among the different members of a family, to confirm which we have collected many examples. Thus an unfortunate suicide writes—"That the distressing characters of a previous attack of insanity, experienced by his wife after a confinement, are so constantly recurring to his mind during her present pregnancy, as never to leave him an instant's calm. He cannot rest day or night, and hardly touches food. He fears they will both go mad, and so prefers death."—One of my friends, a distinguished physician, said to me—"There are times when the erroneous fancies of my wife, with which she is perpetually troubling me, get also a lodging in my own brain, and I am compelled to make an effort to get rid of them." In my establishment I have frequently received both husband and wife in succession, and sometimes the two together.

At other times the writings, though not distinctly stamped with insanity, evidence a morbid exaltation, a romantic exaggeration, or a hypochondriacal disposition of mind.

Insanity may declare itself suddenly and without any premonitory warning. "On getting up," writes a man of business, "I found myself troubled by the blood rushing to my face; at the same time I had a severe pain in the head, then in a moment of exaltation and frenzy, I cut my throat with a razor. The agony and loss of blood restored my reason. I have no longer any wish to kill myself, but if the same symptoms recur, I cannot answer for my acts." Dr. Forbes Winslow relates a similar case in his "Anatomy of Suicide."

Lastly, the disorder of the ideas in the last moments of life may depend on the moral impression of the suicidal act itself, or may arise from the physical operation of the means employed. Many individuals, especially

* Recherches Statistiques sur le Suicide dans la Folie. Annales d'Hygiène, &c. Tome XLII. p. 38, Juillet, 1849.

among the asphyxiated, use some such expressions as the following:—"I am going mad—my brain is on fire—I don't know what I am about—I am lost."

*Recognise their cowardice; express horror of the act; indignation and imprecation against themselves.*—The same difference of opinion which exists among authors concerning suicide, is to be found in the last writings of its victims. Thus, whilst some proclaim it a proof of courage, independence, and stoicism, others declare it criminal, cowardly, and contemptible.

Nine individuals, all males, left letters containing their views on this subject; here are a few fragments:—"I die like a dog, because I could not endure the troubles of life. Why I kill myself is a secret which I keep, but I may state that neither gaming nor women are the cause. Since I spoke to you just now, I have tried four times to blow out my brains with a pistol, and four times has it missed fire. So, to be sure this fifth time, I fire it with a match."—"The loss of all I possessed is the cause of this infamous act; may my death prove a lesson to my son."—"Self-murder is against my principles, but finding ourselves without money and without friends, deprived of all our resources, seeing no chance of work, and unable to meet our engagements, our only refuge is the tomb."—"I know they say it is more courageous to face adversity than to skulk to a grave; but what is one to do at seventy-five years of age? when scoundrels in whom you trusted have swindled you of everything, and you have not even bread to eat? In such a case death may become necessary, or even indispensable."

*Agonies of Mind.*—There is nothing absolute in human affairs. Establish any general proposition, and presently an exception starts up beside it. Thus it has been stated, that all suicides tremble and hesitate at the last fatal moment, and are no longer masters of themselves; that they experience an extreme agitation, and are in some sort bewildered. Now the documents we have collected disprove this assertion, for many of them are written in strong, firm hand, in no way differing from the ordinary calligraphy of the individual, and though composed but a very short time before the fatal deed was committed, betray no signs of trepidation or incertitude of mind. Yet, on the other hand, we have several documents in which the writer's perturbation is evident on the paper, for the characters are so confused and irregular as to be hardly legible. In eight letters (seven men, one woman) the agony of mind produced by the thought of the approaching act, forms a striking contrast with the coolness, calmness, and lucidity of other individuals possessed of stronger self-control. One unfortunate man thus expresses himself:—"The idea of death horrifies me; my brain is burning; it is terrible to kill oneself when full of life. If, in spite of my terrors and despair, I achieve my object, it is because I have no other resource. I have not the courage to write any more." The other letters contain similar expressions.

*Resolve after long hesitation.*—There can be no doubt that in the majority of cases, suicide is the result of long deliberations, and that the fatal moment itself is often attended with an agonizing perplexity. It is not often, however, that suicides record the mental combat which preceded the fatal deed. We have only five letters which refer to the final struggle: "It is after long deliberation, and much trouble and hesitation, that I have taken this sad resolve."—"I made known my misery to an illustrious individual, whose life my father saved, but he did not deign to notice me. Some may find it strange that a man about to leave this world should wish to render an account of his sentiments, or speak of his affairs; but it is a last consolation—a final adieu."—A third says, "I have taken eight days to decide on it."

*Apprehension of Suffering.*—We have not designed this article for statistical information, but with the view of inquiring into the state of the mind of suicides at the supreme moment. Our information is not sufficiently ample or exact to warrant strict statistical deductions. For example, there are only three letters which evince any apprehension on the part of the writers respecting the physical suffering attendant on the contemplated mode of death; yet so small a figure cannot truly represent the number of persons in whom such dread must have existed. And this remark is equally applicable to many of the preceding paragraphs. The following are the principal passages in these three letters:—"I feel the last moment approaching, and I hasten to state that, with the view of avoiding too painful a death, I stuck myself with my knife." He had previously attempted suffocation.—"Yet another hour of horrible suffering, and all will be over."—"Not being able to stand any longer, I cast myself on my bed. I dreaded the sufferings, but did not think they were so severe."

*Fear of Wanting Courage.*—Many persons on the point of destroying themselves are fearful that their determination may fail at the critical moment. Three record this misgiving in their letters. "However strong a man's resolution may be," writes a victim, "and however urgent the necessity, one experiences an extraordinary emotion, in short, a horrible fright, when the moment of execution arrives." In a justicative memoir, an unfortunate official thus expresses himself:—"I was desirous of reforming some revolting abuses, and introducing important improvements in the administration to which I belonged, and by persevering I had succeeded in getting some of them adopted, but those whom my reforms offended cruelly avenged themselves: they overwhelmed me with insult, denounced me, proclaimed me a calumniator, deprived me of my post, and had me shelved. Suicide is my only appeal, and last resource. I shudder at the idea, and my courage fails me; but what should I do on earth filched of my reputation? I have made up my mind." This affair created a painful sensation in the B***** trial.

2nd Sub-section. *Instructions relative to their funeral; Addresses; Concealment of all clues.*—It might be expected that they who kill themselves would care but little what became of their miserable remains; but it is not so. Sixty-seven letters (56 men, 11 women) contain wishes or instructions respecting burial. Some leave a fixed sum, with directions that their funeral may be simple and economical. This request is the most common. Others request their parents, relatives, or friends, to sell the effects they die possessed of, to pay for their interment.

The thought of being hurried off to the last resting-place, unfollowed and unwept, is painful to every mind. Gilbert has expressed this sentiment in some admirable verses—

> "I die, and on the grave towards which I pass
> No friendly tear will fall."

And many entreat that some one will follow their remains, and see them deposited in a grave apart. "If any one will follow my body," says one, "it will soften the horror of my fate."

Sometimes a desire is mentioned to be interred by the side of, or near unto, some beloved person. One letter contains the following:—"Engage my father to follow my funeral, and try to get me buried by the tomb of your mother." On the other hand, a few request that no person may follow them. "If my body is found," writes a man, "bury it without any ceremony. Your presence would be an insult."

Some suicides wish their remains carried at once to the cemetery, with as little fuss and ceremony as possible. Others direct that they shall first be carried to their homes, or to the house of a relative. "I request M. le Commissaire to have my body carried to the house of Mr. B.: his wife, who is my daughter, and for whom I have very little regard, will expect it." These lines were written on a piece of paper enclosed in a bottle, and attached to the chest of the suicide. Several not only give full directions for their funerals, but also leave lists of the persons to be invited, and sometimes write their own epitaphs. A young woman, abandoned by her lover, conjures him to follow her to her last home, hoping, probably, in that manner to inspire him with regret, and awaken a souvenir of his former passion.

Many of those who destroy themselves away from their homes, in localities where they are not known, leave some indication by which they can be recognised. We have 23 papers of this kind: they are commonly addressed to their parents, friends, or acquaintances, and attached to some part of their person or dress. In the instance of suicide by drowning, the indication is sometimes placed in a bottle.

Some few suicides carefully annihilate every trace of their identity; yet even these occasionally leave a writing behind them. One says, "The victim leaves no trace; the executioners will not know her death: why should she tell them that she perished because they refused to relieve her." Indeed, it is painful to think that the negligence and indifference of the authorities in relieving the really destitute is frequently a cause of death. When an unfortunate wretch applies to public charity for a morsel of bread, he should never be sent away without it, for fear that want may render him desperate.

*Instructions as to the mode of their interment.*—One of the last thoughts of the dying is the care of his remains. Very frequently they indicate the place where a sum of money, destined to defray the expenses of their interment, is to be found. This pre-occupation is shown in 24 writings (12 men and 12 women). The following are the chief recommendations:—"I beg that I may be buried in my clothes. . . . . Here are the sheets, the chemise and cap which are destined for this last ceremony. I wish Madame G—— to perform this sad office, and herself place me in the coffin." Twelve individuals, (six men and six women,) who destroyed themselves together in pairs, manifest a wish to be buried in the same shroud. Their letters all contain similar expressions to the following:—"O you, whosoever you may be, do not separate those whom death hath joined; it is our supreme wish, respect it. Let us be buried in the same grave; so that, having lived together on earth, we may rest together in the tomb." A young man conjures the authorities not to separate his remains from those of his sweetheart, adding, that they need not open her body, as she is not pregnant. "We die," adds he, "in each other's arms, kissing one another." Several of these unfortunate couples have, in fact, been found tightly locked in each other's embrace, without any sign of suffering in their features; some, indeed, wore an aspect of joy and contentment.

*Desire to be carried at once to the cemetery.*—It is possible for a person to destroy himself, although fully sensible of the guilt of his action, and in that case a feeling of shame may induce him to direct that his funeral shall be as humble and quiet as possible. Eleven letters (10 men, one woman,) are examples of this feeling. Some request the officials who verify their decease, to inform their friends, and to have them at once carried to the nearest cemetery; others desire to be buried early in the morning, as simply as possible, without any ceremony, mourners, or funeral rites.

*Desire to be buried with some souvenir.*—Occasionally suicides express a wish to be buried with certain articles which they designate, such as a ring, a bracelet, or a portrait. We have 8 letters (5 men, 3 women,) on this head. "I pray," writes one, "to be buried with the hair that I have round my neck; it is my mother's."—"Do not in any way reproach the author of my death," writes a young female, "and in Heaven's name do not take off the bracelet nor the clothes that I have on, but put me in my coffin precisely as I am." A third earnestly requests to be buried in her clothes, and begs that her hair may not be cut.

*Prayer to be buried in the pauper burial-ground.*—If indifference as to the lot of the body after death ever exists, we should look for it among suicides. This is shown in 7 cases, (6 men, 1 woman.) A man thus expresses himself:—"I want no draperies; the pauper's cart and the common hole are my fancy; and, above all, I will not be followed by my hypocritical children, whom I excuse from putting on mourning." In another letter we read—"I wish to be buried like a pauper; until the time comes, my remains may lie in the lesser wing of the mansion in which I have squandered away my fortune." A third writes thus:—"I kill myself voluntarily. I request that I may at once be carried to the cemetery in the pauper's cart, and tossed into the common fosse, like a real outcast. Such are my last wishes. No invitations to my funeral, no mourners, especially let *her* keep away." All direct that their burial should be simple.

*Dread of being exposed at the Morgue.*—However great the indifference shown by most suicides about the fate of their remains, there are some unable to bear the idea of being exposed to the idle gaze of the curious. The Morgue, in particular, is the horror of many, just as are Charenton and Bethlem to countless individuals. Three persons (two men, one woman) beg that they may not be carried to that building.

*Considerations respecting the fate of their remains.*—In opposition to the feeling of indifference about their remains, is the apprehension manifested by some suicides respecting them. A man left on a table, beside his body, this note, without an address:—"My corpse is abandoned; it needs a friendly or compassionate hand to throw over it a few handfuls of earth. My brother, be so good as to undertake this painful office." Another man writes to a friend,—"Do not leave my body here; come and claim it."

3rd Sub-section.—*Regrets at Dying; Vexation at Failure; Indifference.*—The man who destroys himself yields to a temporary madness, or to circumstances more or less pressing, which render life insupportable. There is, then, nothing astonishing in his not expressing regret for his act, nor indeed is it often that any regret is expressed; nevertheless, a certain number manifest sorrow and reluctance at quitting life. This feeling is found chiefly among young persons, and is expressed in 22 letters (20 men, 2 women). "I have lived a happy life for 23 years, and when everything seemed to promise me a fortunate lot, amidst the magnificence of nature, I cast myself into the tomb, seeking to hide myself behind a cold stone slab." Another expresses himself as follows, in a letter to his mistress: "Thy desertion fills me with despair; with thee life flies so happily; my eyes fill with tears at the contemplation of that immense felicity. To live without thee is impossible. I die adoring thee." What a gulf of contradiction is man's heart. The author of the foregoing letter was a bachelor, comfortably situated, whose mistress had left him in a moment of irritation, caused by his obstinate refusal to recognise and adopt their child; and yet, although he declares his mistress is everything to him, even when a word would bring her back, he prefers destroying himself to performing a simple act of justice. "I promised you this morning," writes a young woman, "not to kill myself; but alas! I feel that I have not strength to support life away from you, or to see you pass into the arms of another. It is cruel to die so young, with the thoughts of a happy future before one, but it were a hundred times more cruel to remain alone. Carry this garland to our child's grave; it is the last prayer of her who loves you better than life itself."

In opposition to those regrets at quitting the world, 11 individuals (men) express their disappointment at failing in the attempt. A man and a woman attempt suicide together; the man dies, but the woman is rescued—nevertheless, when perfectly recovered, she continually laments having survived the object of her affection. Some testify that they die satisfied, that they have enjoyed life, and have no regret for the deed they commit.

*Disappointed hopes and expectations.*—To dream is our lot; but alas! what numerous deceptions! No wonder that so many who begin life, full of joyous and brilliant anticipations, get discouraged, and despond when they see all their illusions perish in succession. "What a world I created for myself!" writes a young man of moderate intelligence. "I was young and handsome; glory stood by me to lead me on to fame and fortune; a magnificent prospect was unfolded before my eyes. And now, where am I? In poverty and neglect, misunderstood, wretched—without a soul to notice or take care of me. I have naught left but to die." "I was born under an unlucky star," says another; "and I find that I cannot figure with distinction in life, as I fancied: say that I died of an attack of apoplexy; I don't wish to pass for a coward." A third makes known that he had placed all his hopes of happiness in his union with a woman whom he had long adored; but that after the day for his marriage had been fixed, he discovered her to be pregnant. "I am annihilated," he says; "life is odious to me; and I prefer renouncing it to living with the memory of my lost happiness."

4th Sub-section: *Belief in fatalism.*—For a long time past the consequences of fatalism have been known and appreciated; according to that doctrine there is nothing surprising in suicide, murder, or theft; the crime was prescribed, and its actual occurrence was therefore a matter of course. This pernicious doctrine is frequently the excuse of suicides, and many who destroy themselves say, "such was to be our fate." Nine letters (six men, three women) refer to this belief. A young lady, clever, witty, and accomplished, but destitute of judgment and self-control, whose follies had damaged her reputation and injured her position, writes thus to one of her female friends: "I am a fatalist, and it is my firm conviction that events have a prescribed and unchangeable direction, from which nothing can turn them." A very convenient creed, since it serves to excuse all kinds of folly and crime. Another woman who had trodden under foot all respect for decorum, thus addresses her parents: "I strove to escape my fate, but I have been dragged onwards in spite of myself; an inflexible destiny is the cause of my misfortunes and my death."

5th Sub-section: *Indifference of public opinion.*—When a man resolves to destroy himself he cannot attach much value to public opinion; what is the judgment of his fellow-men to him? And this indifference must be natural to the materialist. We find this sentiment clearly expressed in eight letters (six men, two women). "Say what you will of this deed," writes a young man and his mistress, who destroyed themselves together,

"we care but little; when you speak our hearts will have ceased to beat, and our bodies will be insensible to your insults." An old actor smokes his cigar, calmly bids adieu to his wife, gives his child a piece of barley-sugar, goes into his room and writes these words in pencil, "What is there more natural than to quit when the house is tumbling in pieces. What is there to fear? Opinion! Only fools vex themselves about that." He then walks out, without betraying the least emotion, and throws himself into the Seine.

6th Sub-section:—*Request that their letters may be published in the papers.*—Vanity, which is the distinctive trait of the French character, does not quit us at death; we wrap our mantle about us to die before the public with grace and effect. This passion is abundantly exemplified in the manuscripts we are analyzing. Many of them are manifestly composed to excite pity, sympathy, and interest for the unfortunate writers. Crimes are forgotten, faults and follies are glossed over; the suicide is the victim of adverse circumstances, of the artificial restrictions of society, weak rather than guilty, and always more sinned against than sinning. Such is the sickly sentimentality which some of the first authors of our time have nursed and propagated. A large number of suicides leave letters, which, although the request is not expressly made, are evidently intended for publicity. For it is well known that these letters when interesting are frequently inserted in the journals. We have met with only one document which was accompanied with a special request that it might be published, but, as it contained revelations calculated to compromise many individuals, the magistrates ordered it to be destroyed.

7th Sub-section: *Incertitude about their future destiny.*—The ruling thought at the hour of death must naturally be our future destiny. In vain does man seek to evade the contemplation, to drive out the thought, to invoke oblivion: he feels that all is not over with the last gasp. Doubts and misgivings are revealed in many letters; and is not doubt the first step to belief? One person exclaims—"Do we die entirely, or does our soul appear before God? I am ignorant of what is about to befal me, but there is something within me which tells me, in spite of all my wishes and arguments, that there is beyond the grave a new order of things, which will soon be revealed to me." And this ought to be the belief of every man in that supreme hour, when not deprived of reason, or thoroughly brutalized. "If I have a soul, O my God!" writes a student, "take pity on it, and judge it with all its imperfections."

8th Sub-division: *Frivolous motives.*—Although, in the majority of instances, suicide is the result of violent chagrin, or long-continued physical sufferings, yet, in a small number of cases, it is due to the most frivolous causes. The race of Vatel is not yet extinct. A workman is enraged by his brother taking from him some fried potatoes, and throwing them into the water. In his anger he rushes to strike him, but being withheld and prevented, he suddenly darts off, makes for the Canal St. Martin, throws himself in, and is drowned. We have eleven letters explaining the motives of these bizarre determinations. Here are specimens of some of them:—"My father, I kill myself because you scolded me for not getting more money for the cabriolet."—"I struck myself because I was too fond of gossipping; I wished to punish myself, but I have gone too far."—"To avenge myself on a fellow-soldier, I cut up his accoutrements; but fearing to be punished for it, I have killed myself."—"I so ardently longed to go to the ball, my lover refused to allow me, so I have nothing left but to die."—"A piece of good news which I have heard since I resolved to die, would have made me renounce my project, if I had not already dispatched a letter announcing my suicide."—"I kill myself because I have given my bedfellow the itch."—"I prefer dying to being treated as a blackguard." What a melancholy page in the history of the human heart would that be, which should record the secret trivial causes which so often determine the most important acts of life.

*Resumé.* The analysis of the disposition of mind, in relation to the act of suicide, affords a new proof of the impossibility of too largely generalizing questions of morality, and the hopelessness of finding an universal solution.

The numerous facts adduced in this section show, among other conclusions, that it is possible for man to destroy himself with every mark of *sang froid*, reason, and courage; independently of the suicide's own assertion on that point, it is confirmed—1st, by the letters being written in a clear, firm hand, without blot or erasure, even when bearing the date, "*one hour before death*," by which event they are sometimes interrupted. 2ndly. By the character of the testamentary dispositions, which evince the entire freedom of the intellect, the lucidity of the ideas, and the energy of the will. The exceptions to the preceding facts are a natural consequence of the diversity of man's nature. The mind may be troubled by insanity, a temporary delirium, a momentary exaltation. It is remarkable that insane persons who destroy themselves in asylums very rarely leave any writing behind; whilst insane persons at large frequently detail the motives of their suicide. This shows two things,—first, that there are many unrecognised madmen at large, and, secondly, that the number of suicides really insane is about one-fourth of the total number.*

We find in the sentiments expressed by a certain number of suicides concerning their fatal act, that they treat as blameable, cowardly, and culpable, the diverse opinions of authors.

The humour, disposition, and natural character of individuals singularly modify their sensations; thus the agony of mind in some forms a striking contrast with the self-command of others. Many do not put their resolve into execution until after long hesitation and delay; they dread the suffering, and are fearful of wanting courage, &c.

Considerations relative to burial form the second section. These preoccupy the minds of many: they settle the costs, order the details, designate the persons who are to follow, fix on the place of burial, frequently (especially females) requesting to be interred by the side of some beloved person; others, on the contrary, merely request that their remains may be taken to the nearest cemetery, and thrown into the common fosse. A limited number give minute directions as to what is to be done with their remains, specifying certain objects which they wish to be placed in their coffins. A few show their dread at being exposed at the Morgue, or speculate on the fate of their corpse.

The third sub-section exposes the regrets of some suicides, principally young persons, at quitting life, and the despair of others at having failed in their attempt. Many declare that they have no regret at all.

The fourth sub-section refers to suicides holding, or pretending to hold, fatalist opinions. They say that they could not have acted otherwise, that they have merely obeyed their destiny.

The analysis of the fifth sub-section bears on the indifference which some individuals profess to entertain concerning public opinion on their deed.

In the sixth sub-section we observe the ruling passion of vanity, directing even the suicide, and causing him to aspire to a posthumous notoriety.

* This may be true in France, but we are of opinion that in England the proportion of suicides affected with confirmed or temporary insanity is much greater than one-fourth.—Tr.

The seventh sub-section contains the reflections of suicides on the incertitude of a future life; a momentous question, which must occupy the minds of all at their last hour, and concerning which, reason without faith can afford no certain solution.

The eighth and last sub-section contains the analysis of a series of sentiments, which demonstrate that the most serious actions sometimes proceed from the most trifling causes.

---

### THE MANIAC'S WAIL.

